# The Alum Treatment of Lead Colic

**Published:** 1851-03

**Authors:** 


### RECORD OF MEDICAL SCIENCE.

### PATHOLOGY AND PRACTICE OF MEDICINE.

*The Alum Treatment of Lead Colic.*—M. Brachet, of Lyons, in a recent elaborate prize essay on Lead Colic, presented to the Toulouse Academy of Science, offers very strong testimony, in confirmation of the value of the alum treatment in this disease. Having been medical adviser for above thirty years, to the tinman's trade-union, which of course furnishes a great number of cases of lead-colic, and also physician to a large hospital, he has had under his notice more than 300 cases of the affection. After employing the purgative, opiate, and antiphlogistic systems of treatment, with varied success, M. Brachet finally had recourse to *alum*, as recommended by Gendrin, and was able in 1838, to report highly in its favor, and since that period he has continually employed it, without accident or disappointment occurring. He prescribes from 1½ to 2 drachms in a ptisan, to be taken during the day, adding to this 40 or 50 drops of laudanum, and if the bowels do not act by the third day, giving a mild aperient: and the case is complete. More than 150 patients have been thus treated with entire success, the alum being continued a day or two after the symptoms have disappeared.—*Abridged from British and Foreign Review, January*, 1851.

